# Factors Associated with Health Care Provider Attitudes, and Confidence for the Care of Women and Girls Affected by Female Genital Mutilation/Cutting

**DOI:** 10.1089/heq.2020.0130

**Published:** 2021-05-19

**Authors:** Christina X. Marea, Nicole Warren, Nancy Glass, Crista Johnson-Agbakwu, Nancy Perrin

**Affiliations:** ^1^ School of Nursing, Johns Hopkins University, Baltimore, Maryland, USA.; ^2^ School of Nursing and Health Studies, Georgetown University, Washington, District of Columbia, USA.; ^3^ Arizona State University Southwest Interdisciplinary Research Center, Phoenix, Arizona, USA.

**Keywords:** female genital cutting, female genital mutilation, female circumcision, health care provider, attitudes, confidence

## Abstract

**Background:** Female genital mutilation/cutting (FGM/C) is a cultural practice that includes procedures that intentionally alter or cause harm to female genital organs for nonmedical reasons, affecting ∼200 million women and girls globally. Health care providers in the United States often lack confidence to provide appropriate FGM/C-related care, and experience attitudes that may negatively impact quality of care for FGM/C.

**Methods:** We conducted a cross-sectional survey of health care providers to explore the associations between health care provider characteristics, awareness of health complications of FGM/C, attitudes, and confidence for FGM/C care.

**Results:** Factors associated with more *Confidence for Clinical FGM/C Care* include awareness of health complications, ever cared for a woman with FGM/C, being a woman or person of color, and more than 5 years of clinical practice. Increased *Confidence in Communication Skills for FGM/C Care* was associated with awareness of more health complications for FGM/C. Women endorsed significantly less *Negative Attitudes* toward FGM/C compared with men; no other factors were associated with health care provider attitudes.

**Conclusion:** Future research should further investigate factors associated with health care provider attitudes toward FGM/C and those affected by the practice to promote quality care. Health providers require adequate training for clinical FGM/C care and in the communication skills that promote patient/provider communication cross-culturally.

**Trial Registration:** Clinical Trials.Gov ID no. NCT03249649, Study ID no. 5252. Public website: https://clinicaltrials.gov/ct2/show/NCT03249649

## Introduction

Female genital mutilation/cutting (FGM/C) includes procedures that intentionally alter or cause harm to female genital organs for nonmedical reasons, and affects ∼200 million women and girls globally.^1^ FGM/C is practiced in ∼30 countries with the majority in sub-Saharan Africa, and others in the Middle East and South Asia.^2,3^ Although FGM/C prevalence rates are falling globally, the number of girls and women affected is expected to rise in the coming decades because of persistently high fertility rates in FGM/C practicing countries.^3^ The COVID emergency is currently contributing to a rise in cutting in some regions, with an estimated additional 2 million girls at risk for FGM/C.^4^

In the United States, the Centers for Disease Control (CDC) estimates that 545,000 women and girls may have undergone FGM/C or been born to women from FGM/C-practicing countries.^5^ This latter group is often assumed to be at-risk for FGM/C. Health care providers in countries where FGMC is not normative are increasingly likely to care for affected women and girls due to global migration trends and must be able to meet the health care needs of this group.^3^

FGM/C is conducted on girls between infancy and adolescence, usually by the age of 15, and may include practices from symbolic nicking of the clitoris to infibulation (cutting and sewing a narrowed vaginal opening) depending on the region and cultural group.^3^ FGM/C is primarily conducted by nonmedical providers, such as traditional birth attendants, although there is a trend toward medicalization (when FGM/C is performed by a health care provider).^6^ All types of FGM/C have been associated with adverse health consequences, including immediate, gynecologic, obstetric, and mental and sexual health outcomes; however, not all women with FGM/C will experience adverse health effects, and more severe morbidity is associated with more extensive forms of cutting.^7,8^

Women and girls who have experienced FGM/C require specialized health care to address the possible health complications.^8–14^ For women with type 3 FGM/C, *defibulation,* or the surgical release of the FGM/C scar to widen the vaginal opening, is an important intervention that can lessen or eliminate some health complications of FGM/C and prevent some complications of childbirth associated with FGM/C.^15^ However, health care providers rarely receive training for the care of women and girls who have experienced FGM/C,^16–21^ and obstetric providers rarely receive training to perform defibulation.^22^ Those providers who have received training often report that they would benefit from additional training.^18,23–25^ A recent survey of obstetric providers in the United States found that ∼30% would perform reinfibulation, a type of FGM/C that includes the partial or complete resuturing of the vulva following defibulation, if a woman requested it.^22^ In the United States and other Western countries, health care providers may find themselves facing ethical dilemmas as they balance an opposition to FGM/C as a practice with adult women's right to bodily modifications.^26^

Although there are guidelines available for the care of women and girls affected by FGM/C from the World Health Organization (WHO), and professional and advocacy groups, the health outcomes and experiences of FGM/C affected populations receiving care in the diaspora do not reflect high-quality care.^7,27^ The WHO defines quality care as being effective, efficient, accessible, patient-centered, equitable, and safe.^28^ Women living with FGM/C experience excess cesarean birth rates for nonobstetric reasons.^29,30^ A recent meta-synthesis of qualitative studies exploring the birth experiences of migrant women living FGM/C finds that they report fear of and a lack of trust in their health care providers.^31^ Somali women living with FGM/C in Ohio report experiencing barriers to care that result in delays, and are less likely to access preventative health services.^32^ Somali women living with FGM/C in the United States report experiencing disrespect and stigma in the health care setting.^33^ A qualitative study of women living with FGM/C in Boston found that they feel reluctant to report FGM/C, or health complications associated with FGM/C, because of the negative attitudes of health care providers, or because they may not realize the symptom may be related to their FGM/C status.^34,35^

The lack of health care provider awareness about the health consequences of FGM/C further degrades the quality of care.^36^ To provide quality care for FGM/C, health care providers must be aware of the potential health complications of FGM/C, be confident in their ability to manage care for women and girls who have experienced FGM/C, and understand how their own attitudes toward FGM/C and those affected by the practice may impact how they provide care.

### Conceptual approach

The *knowledge, attitudes, and practices (KAP)* framework is often used to assess health care providers who care for women and girls affected by FGM/C.^16^ The KAP framework theorizes that an individual learns about a topic (knowledge), develops some affective response (attitude), and engages in a behavior (practice)—often these factors influence one another in multidirectional ways.^37^ Existing studies assessing health care providers' KAP have typically reported their results as purely descriptive, without exploring the relationships between knowledge, attitudes, and practices or considering health care provider characteristics, such as demographics or past experiences with FGM/C, as confounders to these relationships.^16,17^

The purpose of this study was to explore the relationship between provider characteristics, including awareness of the health complications of FGM/C (knowledge), and their attitudes toward FGM/C and confidence in their ability to care for patients affected by FGM/C (practice). Self-reported confidence is a proxy for practice when we cannot directly observe provider care.^38^ A rigorous examination of the relationship between provider characteristics, awareness, attitudes, and confidence will provide direction for future FGM/C-related training.

## Methods

### Study setting

We conducted an online cross-sectional survey of health care providers at the time of registration in a workshop titled “Optimizing Care for Women and Girls Affected by FGM/C” in the Greater Phoenix and Tucson, Arizona, and Baltimore, Maryland areas.

### Recruitment and study population

Health care providers were invited to register for the workshop and complete the survey via emails that were distributed to list-servs at 14 health care institutions in Phoenix and Tucson, Arizona metropolitan areas, and distributed to the Johns Hopkins Health System and Johns Hopkins University Schools of Medicine, Nursing and Public Health, as well as to professional organizations in the Greater Baltimore, Maryland, and Washington D.C. area. List-serv contacts included nursing and residency training program directors, medical directors, nursing and medical faculty, and hospital department chairs, and points of contact for local chapters of professional organizations such as Association of Women's Health, Obstetric and Neonatal Nurses (AWHONN), American College of Nurse Midwives (ACNM), and American College of Obstetricians and Gynecologists (ACOG). Electronic consent was obtained from all participants.

The Greater Baltimore area is home to large populations of migrants from Sudan, Ethiopia, and Eritrea, while Arizona has received a large number of Somali refugees.^39,40^ These countries have high FGM/C prevalence (74–98%), and FGM/C type in these countries tends to be type 3—the most extensive form of cutting with the highest rate of mordibity.^3^ The study population for this analysis includes physicians, residents, nurse-practitioners, and nurse-midwives who care for women or girls and are in current clinical practice at least 1 day per month. We excluded nurses, health professional students, mental health, and social work providers.

### Measures

The online questionnaire included four sections: provider characteristics, awareness of health complications of FGM/C, attitudes toward FGM/C, and confidence in providing care for women with FGM/C. We measured awareness of health complications of FGM/C using a 33-item checklist that comprised health complications identified by the 2016 WHO Guidelines.^7^

The attitudes and confidence measures were developed by our research team that includes clinical and research experts in FGM/C. We validated the measures using exploratory factor analysis. The development and psychometric validation of the measures are presented in a separate article currently available as a preprint.^41^ The Attitudes measure includes two subscales “Negative Attitudes toward FGM/C and Those Affected by the Practice” (referred to henceforth as *Negative Attitudes* scale) and “Empathetic Attitudes toward FGM/C and Those Affected by the Practice” (*Empathetic Attitudes* scale). The Attitudes measure includes items that assess attitudes toward FGM/C, and those affected by the practice including women, families, and communities. The Confidence measure includes two subscales *Confidence in Clinical FGM/C Care* and *Confidence in Critical Communication Skills for FGM/C Care*. The Attitudes and Confidence scales both have Likert response options from 1=strongly disagree, 2=disagree, 3=agree, and 4=strongly agree. See Table 1 for sample items and Cronbach's alphas.

**Table 1.** Attitudes and Confidence Scale Characteristics and Sample Items

Scale names and example items	Cronbach's alpha	Number of items
Negative attitudes toward FGM/C and those affected by the practice	0.814	5
Health care providers who perform any form of FGM/C, including symbolic nicking, should be charged with a crime		
Empathetic attitudes toward FGM/C and those affected by the practice	0.628	5
Symbolic nicking or cutting of the female genitalia is an effective way to reduce the harm of FGM/C compared with more extensive procedures		
Confidence in clinical FGM/C care (five items)	0.857	5
On inspection of the female genitalia, I can identify a woman with FGM/C		
Confidence in critical communication skills for FGM/C care	0.694	3
Respond to the health concerns of women with FGM/C by engaging in nonjudgmental listening		

FGM/C, female genital mutilation/cutting.

### Statistical analysis

We performed the statistical analysis using SPSS (version 26). We addressed missing data in scale scores using replacement for the average for any participants who had completed at least 75% of the items for the five-item scales, and 66% of items for the three-item scale. This method of imputation may reduce variability in the data and weaken correlation estimates; however, it does allow us to utilize all cases for analysis. Descriptive statistics are presented as count and percentages. We used multivariable linear regression to explore the association of participant characteristics, previous clinical FGM/C experiences, and awareness of health complications of FGM/C with attitudes and confidence.

### Ethics statement

We received approval from the Arizona State University and Johns Hopkins Medical Institute Institutional Review Board (IRB).

## Results

### Participant characteristics

A total of 796 health care providers attended training events in Arizona and 101 in Maryland for a total of 897 possible survey participants. A total of 354 health care providers completed the online survey (response rate 39.5%), of whom 164 respondents met the inclusion criteria. Study participants were predominantly physicians (28%) or medical residents (48.8%), female (73.2%), and white (76.8%). About half the sample specialized in women's health (47%), and the majority had <5 years of clinical experience (62.2%). See Table 2 for detailed participant characteristics. Most participants had previously cared for a patient with FGM/C (65.9%), although less than half had received training in how to care for women affected by FGM/C (41.5%). See Table 3 for detailed participant clinical FGM/C experiences. There were no significant differences in demographics or FGM/C experiences by site.

**Table 2.** Participant Characteristics (*n*=164)

	Combined, *n* (%)
Clinical practice	
Outpatient medical care	
Resident	80 (48.8)
Physician	46 (28.0)
CNM	28 (17.1)
NP	10 (6.1)
Gender	
Female	120 (73.2)
Male	33 (20.1)
Missing/declined/other/trans	11 (6.7)
Race/ethnicity	
Person of color	37 (22.6)
Latino/Hispanic	11 (6.7)
Asian	16 (9.8)
Black/African American/Native American/other nonwhite^a^	10 (6.1)
White	126 (76.8)
Missing/declined	1 (0.6)
Women's health specialty	
Yes	77 (47.0)
No	79 (48.2)
Missing	8 (4.9)
Scope of practice includes BIRTH (Ob/Gyn, midwife)	
Yes	76 (46.3)
No	80 (48.8)
Missing	8 (4.9)
Years in practice	
<5	102 (62.2)
5–10	22 (13.4)
10–20	16 (9.8)
>20	23 (14.0)
Missing/declined	1 (0.6)

^a^ Due to small *n* in these groups, they were collapsed to protect participant confidentiality.

CNM, Certified Nurse Midwife; NP, nurse practitioner.

**Table 3.** Participant Experiences with Female Genital Mutilation/Cutting (*n*=164)

	Combined, *n* (%)
Ever cared for a patient with FGM/C	
Yes	108 (65.9)
No	56 (34.1)
Previous FGM/C training	
Yes	68 (41.5)
No	96 (58.5)
Aware of defibulation?	
Yes	105 (64.0)
No	59 (36.0)

### Factors associated with health care provider attitudes and confidence for FGM/C care

First, we ran descriptive statistics for the four subscales. The *Negative Attitudes, Empathetic Attitudes,* and *Confidence for Clinical FGM/C Care* subscales have a possible range from 5 to 20, with higher scores indicating more negative attitudes, more empathetic attitudes, or higher level of confidence, respectively. The *Confidence in Critical Communication Skills for FGM/C Care* subscale has a possible range of 3–12, with higher scores indicating a higher level of confidence. Descriptive statistics for the scores on the Attitudes and Confidence scales are presented in Table 4.

**Table 4.** Attitudes and Confidence Scales—Descriptive Statistics

	*n*	Mean	Standard deviation	Minimum	Maximum	Possible range
Health care provider attitudes toward FGM/C and those who practice FGM/C						
Negative attitudes toward FGM/C and those who practice	154	16.21	2.40	10	20	5–20
Empathetic attitudes toward FGM/C and those who practice	150	11.28	2.33	5	20	5–20
Health care provider confidence for the care of women affected by FGM/C						
Confidence for clinical FGM/C care	155	11.38	2.98	5	20	5–20
Confidence in critical communication skills for FGM/C care	157	9.02	1.44	3	12	3–12

Next, we explored factors associated with increased health care provider confidence for the care of women affected by FGM/C. Factors associated with increased health care provider scores on the *Confidence for Clinical FGM/C Care* include being aware of more health complications of FGM/C, having ever cared for a woman affected by FGM/C, identifying as female, identifying as a person of color, and having more than 5 years of clinical experience. Neither having received previous training for FGM/C nor being a women's health care provider was significantly associated with higher scores for *Confidence for Clinical FGM/C Care.* The only factor significantly associated with higher scores on the *Confidence in Critical Communication Skills* scores was awareness of more health complications of FGM/C. See Table 5 for detailed analysis of factors associated with health care provider confidence.

**Table 5.** Factors Associated with Health Care Provider Confidence—Multivariable Analysis

	Confidence for clinical FGM/C care (n=139)^a^			Confidence in critical communication skills for FGM/C (n=140)^a^		
	B (S)	95% CI	*p*	B (S)	95% CI	*p*
Awareness of health complications	0.265	0.047 to 0.140	***<0.001***	0.187	0.002 to 0.059	***0.035***
Women's health provider	0.089	−0.365 to 1.389	0.249	0.074	−0.333 to 0.733	0.459
Ever cared for a woman affected by FGM/C	0.340	1.145 to 3.103	***<0.001***	0.142	−0.181 to 1.002	0.172
Ever received training for care of women affected by FGM/C	0.066	−0.408 to 1.182	0.338	−0.012	−0.515 to 0.450	0.894
Female gender	0.178	0.320 to 2.265	***0.010***	−0.110	−0.625 to 0.559	0.755
Person of color	0.161	0.242 to 2.029	***0.013***	0.026	−0.459 to 0.631	0.755
More than 5 years of clinical experience	0.135	0.037 to 1.607	***0.040***	0.034	−0.383 to 0.571	0.696

Bold-italic signifies statistically significant findings.

^a^ Participants who were missing one or more of the predictor variables were excluded from the analysis.

B, beta; CI, confidence interval; S, standardized.

Next we explored participant characteristics associated with attitudes toward FGM/C and those affected by the practice. Women had significantly lower scores on the *Negative Attitudes* scale compared with men. No other factors were significantly associated with negative attitudes scores. We did note that those who had ever received training for FGM/C care and those who identify as a person of color tended to have lower scores on the Negative Attitudes scale than their counterparts; however, none of these was significant. Only one variable (more than 5 years of clinical experience) was significantly associated with scores on the *Empathetic Attitudes* scale, demonstrating that those with more experience report less empathetic attitudes. See Table 6 for detailed results of the multivariable regression.

**Table 6.** Factors Associated with Health Care Provider Attitudes—Multivariable Analysis

	Negative attitudes (n=138)^a^			Empathetic attitudes (n=134)^a^		
	B	95% CI	*p*	B	95% CI	*p*
Awareness of health complications	0.037	−0.039 to 0.060	0.674	0.067	−0.031 to 0.070	0.452
Women's health-focused clinician	0.033	−0.790 to 1.093	0.751	0.051	−0.734 to 1.219	0.624
Ever cared for a woman affected by FGM/C	0.084	−0.618 to 1.445	0.429	0.088	−0.618 to 1.510	0.409
Ever received training for care of women affected by FGM/C	−0.160	−1.589 to 0.092	0.080	0.119	−0.310 to 1.455	0.201
Female gender	−0.234	−2.380 to −0.332	***0.010***^a^	0.060	−0.727 to 1.448	0.513
Person of color	−0.109	−1.586 to 0.337	0.201	0.067	−0.598 to 1.376	0.437
More than 5 years of clinical experience	0.036	−0.654 to 1.005	0677	−0.180	−1.776 to −0.039	***0.041***

Bold-italic signifies statistically significant findings.

^a^ Participants who were missing one or more of the predictor variables were excluded from the analysis.

### Defibulation/reinfibulation

We performed descriptive analyses of items related to defibulation and reinfibulation among health care providers who attend births, including obstetrician/gynecologists, obstetrician/gynecologist residents, and nurse-midwives (*n*=76). Only 8 (10.5%) providers responded that they had been trained to perform defibulation. Almost half of those who attend births (42.1%) agreed or strongly agreed that heath care providers should perform reinfibulation if the woman requests it. Only a third of health care providers who attend births agreed or strongly agreed that they can perform defibulation during the second stage of labor (35.3%). Fewer than half of the health care providers who attend births (45.9%) agreed or strongly agreed that they could respond to a request for reinfibulation with cultural humility.

## Discussion

This study provides the first exploration of the relationship between health care provider characteristics, awareness of health complications, attitudes toward FGM/C, and confidence for FGM/C care using psychometrically validated scales. Existing studies assessing health care providers caring for women and girls affected by FGM/C tend to report descriptive findings, without exploring how these factors are interrelated. Our study sample includes a diverse cross section of health care providers including physicians, nurse-practitioners, and nurse-midwives who practice in regions with considerable numbers of refugees and immigrants from regions where FGM/C is common. Of the providers we surveyed, two-thirds had ever cared for a patient with FGM/C, but fewer than half had received any training for the care of those affected by FGM/C. This is consistent with a recent U.S. survey of obstetric providers, which found that 56% has received some didactic and 26% hands-on clinical training, and 60% had ever cared for someone with FGM/C.^22^

Participants reported moderate levels of confidence for the clinical care of patients living with FGM/C. This is consistent with the findings of an existing qualitative synthesis that found health care providers are often unsure of what constitutes appropriate care for those affected by FGM/C, and many desire additional training.^42^ Interestingly, having received prior training for FGM/C was not significantly associated with increased *Confidence for Clinical FGM/C Care*. This may indicate that existing training interventions are inadequate, and do not provide the opportunity for health care providers to achieve competence before caring for patients. Simulation-based training may be an effective modality for FGM/C-related care because it has been demonstrated to improve health care provider confidence and positively affect patient outcomes, particularly for care of a relatively uncommon condition such as FGM/C.^43^

Participants in our study reported high levels of confidence in their communication skills. This is a more surprising finding in the context of existing studies of health care providers who note their frustration with cross-cultural communication and lack of confidence with interpreter use.^44^ Research with women and girls living with FGM/C demonstrates that they often feel disrespected and stigmatized by their providers, and thus, provider confidence may be misplaced.^31^ The only factor associated with increased confidence in communication was awareness of more complications associated with FGM/C, suggesting that the first step toward increasing provider confidence is increasing their knowledge about FGM/C and its consequences.

Future research should explore how patients and providers interpret and experience communication during clinical visits to identify areas of incongruence. Research studying patient/provider communication, particularly in the presence of racial, cultural, and/or linguistic discordance, has demonstrated that health care providers often experience implicit bias that is transmitted to the patient through their communication behaviors.^45,46^ Health care providers caring for women who have experienced FGM/C in the diasporic setting may be further influenced by “othering” of African bodies, and moral superiority of opposition to FGM/C may lead to a paternalistic and stigmatizing treatment of women living with FGM/C.^47^

Our findings were limited in terms of factors associated with the *Negative and Empathetic Attitudes* subscales. Only one factor had a significant association—identifying as a woman was significantly associated with less negative attitudes toward FGM/C compared with identifying as a man. Women also had significantly higher scores on the *Confidence for Clinical FGM/C Care* scale. A study conducted in Spain also found significant gender differences; women were more likely to detect FGM/C cases and correctly identify FGM/C, while men were more likely to include reporting women with FGM/C to the authorities as part of their response.^23^ No other variables under investigation were significantly associated with scores on the Attitudes subscales.

These scales were developed for use in this study, and likely require further refinement including potentially the inclusion of additional items to broaden the range of attitudes assessed. Furthermore, our sample was self-selected and so may have less variance in terms of the attitudes compared with a random sample of health care providers. Finally, future research should consider investigating the association between health care provider attitudes scores and factors such as scores on a validated measure of implicit bias, and/or political affiliation, which may inform attitudes toward immigrants in our current highly politicized anti-immigrant environment.^48^

The Attitudes scales that we developed were designed to be used with any health care provider, regardless of scope of practice, while the Confidence scales were designed for any health care provider who provides outpatient care. Women with FGM/C require specialized consideration from all health care providers; however, there are important skills for providers who attend birth. It is concerning that only about 10% of providers who attend births have been trained to perform defibulation, an important intervention for reducing obstetric morbidity.^15^ Despite only 10% reporting receiving training for defibulation, about a third agreed or strongly agreed that they are confident that they could perform defibulation during the second stage of labor. This may represent an overconfidence on the part of providers, and a risk to women with FGM/C.

We found that almost half of the providers who attend births agree or strongly agreed that a provider should perform reinfibulation if the woman requests it. This is a controversial stance given that reinfibulation is considered a form of FGM/C, and thus vehemently opposed by the WHO. This is not completely surprising given that no professional health organization has published FGM/C-specific guidelines in more than a decade. Reinfibulation is associated with similar health complications as other forms of FGM/C. However, there is a dearth of research on the consequences of *partial* defibulation and *partial* reinfibulation, which may have different outcomes related to possible physical health complications or mental health and well-being, particularly genital self-image or bodily satisfaction.^47^ An important difference is that reinfibulation is typically performed on an adult woman who can legally consent to the procedure. In many high-income countries in the West, medical ethicists agree that adults have the right to bodily modifications that are without direct medical benefits including cosmetic genital surgery.^49,50^

While the ethics of this debate are beyond the scope of this article, it is important that health care providers receive adequate training regarding the ethical dilemmas they may face during the provision of care so that they are not surprised by a request, and that they may have a considerate and respectful response should a request arise.

## Conclusion

Few health care providers receive any training for the care of women and girls who have experienced FGM/C, and those who have received some training are not necessarily more confident in their ability to provide appropriate clinical care for FGM/C. The overall negative attitudes toward FGM/C and those affected by the practice may be consistent with the overall discriminatory attitudes toward patients of color in the United States. Given the gross disparities in maternal and neonatal outcomes by race, strategies to help providers recognize and mitigate their negative attitudes are imperative. The high level of willingness to perform reinfibulation paired with a lack of understanding and training on how to perform defibulation or manage a vulvar scar highlights the need for more explicit guidelines for U.S. providers.

Guidelines should include a structure for providers to explore their attitudes regarding reinfibulation, obtain appropriate training for defibulation and reinfibulation, and guide providers in how to have a culturally informed discussion with patients about the health and ethical issues related to a woman's choice.

Our research has demonstrated innovative and important opportunities for the development of future education and training for health care providers caring for women and girls affected by FGM/C. Specifically, future training for health care providers should include opportunities to practice clinical and communication skills through structured clinical simulations, which are more effective than didactic teaching for building health care provider confidence. Training should also include opportunities for discussion and reflection of individual attitudes toward the practice of FGM/C and those who are affected by the practice. Further research should explore how simulations and structured discussions around the power dynamics of providing care to marginalized and oppressed groups can further transform attitudes, confidence, and quality of care.

## Abbreviations Used

ACNM: American College of Nurse Midwives
ACOG: American College of Obstetricians and Gynecologists
AWHONN: Association of Women's Health, Obstetric and Neonatal Nurses
B: beta
CDC: Centers for Disease Control
CI: confidence interval
CNM: Certified Nurse Midwife
FGM/C: female genital mutilation/cutting
IRB: Institutional Review Board
KAP: knowledge, attitudes, and practices
NP: nurse practitioner
S: standardized
WHO: World Health Organization
